# Medical and dental students’ attitude and practice of prevention strategies against hepatitis B virus infection in a Nigerian university

**DOI:** 10.11604/pamj.2017.28.33.11662

**Published:** 2017-09-14

**Authors:** Oyebimpe Jumoke Adenlewo, Peter Olalekan Adeosun, Olawunmi Adedoyin Fatusi

**Affiliations:** 1 Faculty of Dentistry, Obafemi Awolowo University, Ile-Ife, Nigeria; 2 Dental Hospital, Obafemi Awolowo University Teaching Hospitals Complex, Ile-Ife, Nigeria

**Keywords:** Medical and dental students, hepatitis B vaccine, universal precaution, Nigeria

## Abstract

**Introduction:**

Medical and dental students are a high-risk group for hepatitis B virus (HBV) infection which is an occupational hazard for them and a leading cause of death globally. Prevention strategies include vaccination and observance of standard precaution. However, available reports claim utilization of the prevention strategies is low. This study evaluated the attitude of the students towards HBV vaccine and cross-infection practices.

**Methods:**

This study was a cross-sectional study carried out at the College of Health Sciences, Obafemi Awolowo University, Nigeria. Using the convenience sampling method, anonymous self-administered questionnaires were distributed to the first 120 participants that volunteered to participate in the study. Data analysis was done using IBM's Statistical Package (SPSS) version 20 software. Statistical level of significance was set at p < 0.05.

**Results:**

Over eighty percent (83.2%) of the participants had at least a dose of the HBV vaccine while 79.65% completed the three doses. Majority (94.7%) of the students that did not receive the vaccine cited their busy schedule as the reason for their failure to be vaccinated. Taking every patient as a contagious disease risk (86.5%), washing hands after contact with patients' body fluids (82.1%) and wearing gloves before touching mucous membranes and non-intact skin (74.1%) were the most practiced universal standard precaution items.

**Conclusion:**

The uptake rate of HBV vaccination and practice of standard precaution among the students are commendable. However, there is need for improvement considering the level of HBV infection in Nigeria.

## Introduction

Hepatitis B virus (HBV) infection is one of the major health concerns worldwide with a global incidence of approximately 4.5 million cases per annum and a highly lethal disease causing approximately 620,000 deaths per annum globally [1]. The situation is worse in Africa which is classified as a high-endemic region, only second to Asia in prevalence rate. In sub-Saharan Africa, at least one HBV marker will be found in 70-75% of adults and in estimations done in West Africa, 40% of children will be infected by the age of two years and more than 90% by the age of ten years [2]. The HBV is a partially double-stranded DNA virus that belongs to the family, hepadnaviridae. Four major serotypes, listed as adw, ayw, adr and ayr have been identified and at the level of the surface antigen (HbsAg), nine minor subtypes have been identified [3]. Following the complete sequencing of the HBV DNA from different parts of the world, eight genotypes (listed as A-H) and several subgenotypes with distinct geographical distribution have been identified [1].

Acute HBV infection typically resolves spontaneously or progresses to chronic infection [4]. Traditionally, chronic HBV infection goes through three phases namely, an immune-tolerant phase, an immune-active phase and an inactive phase, which is the carrier phase characterized by little or no clinical disease. Patients can move progressively from one phase to the other or revert backwards [5]. Some patients may undergo spontaneous resolution of disease after the third phase or undergo a fourth phase which some authors have described as the reactivation phase [4, 6, 7]. A number of factors determine the progress of patients through these phases. Such factors include level of HBV endemicity in the sufferer's community, sufferer's age at the time of acquisition of infection, mode of transmission and HBV genotypes and subgenotypes [8]. Hepatocellular carcinoma and liver cirrhosis are both fatal conditions that are primary outcomes of chronic HBV infection [5]. HBV is responsible for 80% of liver cancer [2].

The HBV is transmitted by contact with infected blood or body fluids such as semen, urine and saliva [4, 9,10]. Healthcare workers (HCW's), especially in a developing country like Nigeria, are at a higher risk of HBV infection, making its nosocomial transmission of a great significance [11]. The mainstay of prevention of HBV transmission is the practice of standard precautions and vaccination [12]. Standard precautions include such activities as hand hygiene, proper disposal of sharps and wearing of personal protective equipment (gloves, gowns, goggles, cap wearing) [13]. HBV infection is an occupational hazard for HCW's as those who are unimmunized are at risk of contracting the virus in their workplace [14, 15]. Unfortunately, uptake of the HBV vaccine among doctors and dentists in a developing country like Nigeria is low [16–18]. Students in clinical training are also exposed to risks of HBV transmission via contact with patients' body fluids and needlestick injuries and their rate of exposure is comparable to that of hospital staff [19]. It has been established that Nigerian students in clinical training are at a great risk of HBV infection [20]. This study investigated the attitude toward the uptake of the HBV vaccine and the practice of standard precaution among clinical medical and dental students of the Obafemi Awolowo University, Ile-Ife, Nigeria.

## Methods

This questionnaire-based descriptive cross-sectional survey was conducted at the College of Health Sciences, Obafemi Awolowo University, Ile-Ife, Nigeria. Ethical clearance was sought from the Health Research Ethics Committee of the Institute of Public Health in the College of Health Sciences and clinical students (years four to six) in medicine and surgery and dentistry were recruited into the study. After informed consent was obtained from willing participants, well-structured questionnaire was used to obtain their sociodemographic data and HBV vaccination status. If they were not yet vaccinated, the reasons why they were not vaccinated were also obtained. Their practice of standard precaution was also evaluated using Likert scales. Data analysis was done using IBM's Statistical Package (SPSS) version 20 software. Statistical level of significance was set at p < 0.05.

## Results

**Figure 1:**
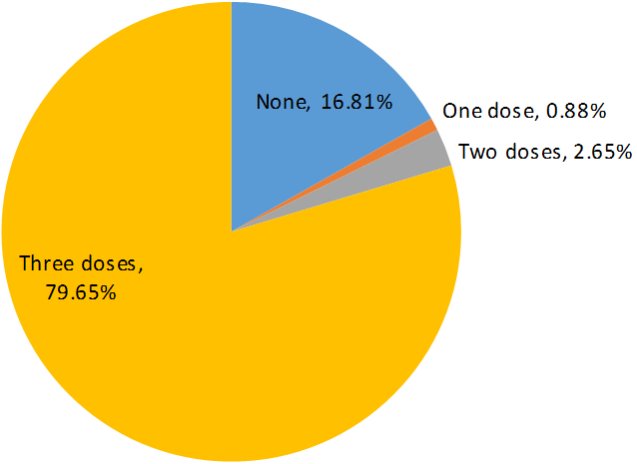
Rate of vaccination rate

A total of 120 questionnaires were administered and 113 (94.2%) were correctly filled and returned. The mean age of study participants was 24.97±2.09 years (range: 20-30 years). Table 1 shows the sociodemographic data. Seventy-six (63.3%) of the students were aged between 23-26 years while 71 (59.2%) were males. A higher proportion of females (95.24%) more than males (76.06%) received the vaccine, a higher proportion of dental students (88.71%) more than medical students (76.47%) received the vaccine while the clinical III students had the highest proportion (86.11%) of vaccine uptake. However, the differences in vaccine uptake for all the parameters were not statistically significant. Clinical III students constituted 95.6% of the entire participants and this can account for the disproportionate representation of the Clinical III students among the vaccine uptakers. Ninety-four (83.2%) of the students took at least a dose of the vaccine. Ninety students (79.65%) completed the three doses (Figure 1) and constituted 95.7% of all those that started the vaccination exercise. In other words, 19 (16.8%) of the students did not even attempt vaccination and only four (4.3%) of the students that attempted vaccination failed to complete it (Table 2).

**Table 1:** Demographic characteristics and vaccine uptake

Sociodemographic characteristics		Vaccine uptake		
Age (yrs)	Yes (%)	No (%)	Total (%)	Χ^2^ (*p*-value)
19 – 22	11 (91.67)	1 (8.33)	12 (100)	
23 – 26	62 (81.58)	14 (18.42)	76 (100)	0.769 (0.681)
27+	21 (84.00)	4 (16)	25 (100)	
**Gender**	**Yes**	**No**	**Total**	
Male	54 (76.06)	17 (23.94)	71 (100)	
				6.942 (0.08)
Female	40 (95.24)	2 (4.76)	42 (100)	
**Course of Study**	**Yes**	**No**	**Total**	
Medicine and Surgery	39 (76.47)	12 (23.53)	51 (100)	
				2.997 (0.83)
Dentistry	55 (88.71)	7 (11.29)	62 (100)	

**Table 2:** Rate of completion among students who attempted vaccination

Number of doses	Frequency	Percentage (%)	Cumulative Percentage (%)
One	1	1.1	1.1
Two	3	3.2	4.3
Three	90	95.7	100.0
Total	94	100.0	

Among the 16.8% of the participants that never received the vaccine, a vast majority of them (94.7%) cited their busy schedule as the sole reason or one of the reasons for their failure to receive the vaccine. Only 5.3% believed that the vaccine was unavailable (Table 3). Most participants always wore gloves before touching mucous membranes and non-intact skin (74.1%), washed hands after contact with patients' body fluids (82.1%) and took every patient as a contagious disease risk (86.5%) (Table 4). There were differences between the proportions of the medical and dental students who always practiced standard precaution but the only activities with statistically significant differences between these two groups were wearing goggles while treating patients (p = 0.046), wearing face masks while treating patients (p < 0.001) and taking every patient as a contagious disease risk (p = 0.017). Medical students tended to wear goggles while treating patients more than their dental counterparts while dental students tended to wear facemasks while treating patients and took patients as contagious disease risks more than their medical counterparts (Table 5).

**Table 3:** Reasons for failure to receive the vaccine

Reason	Frequency	Percentage (%)	Cumulative Frequency (%)
Vaccine unavailable	1	5.3	5.3
Busy schedule	11	57.9	63.2
Vaccine unavailable and busy schedule	2	10.5	73.7
Distant vaccination centres, Vaccine unavailable and busy schedule	4	21.1	94.7
Distant vaccination centres, busy schedule and did not know about the vaccination processes	1	5.3	100.0
Total	19	100.0	

**Table 4:** Practice of standard precaution

	**Always**Percentage(%)	**Frequently**Percentage(%)	**Sometimes**Percentage(%)	**Rarely**Percentage(%)	**Never**Percentage(%)
Wear gloves before touching membranes and non-intact skin	74.1	13.4	9.8	2.7	0.0
Wear goggles while treating patients	8.0	7.1	8.0	33.0	43.8
Wear protective gowns while treating patients	68.8	18.8	6.3	3.6	2.7
Wear protective masks while treating patients	46.9	11.5	18.6	11.5	11.5
Discard sharps into a waste container after treating patients	31.8	10.0	3.6	10.9	43.6
Wash hands before and after treating patients	61.1	29.2	9.7	0.0	0.0
Wash hands after contact with patients’ body fluids	82.1	17.0	0.9	0.0	0.0
Take every patient as a contagious disease risk	86.5	8.1	5.4	0.0	0.0

**Table 5:** Practice of standard precaution and course of study

% of ‘Always’ on the Likert scale			
	Medicine and Surgery	Dentistry	X^2^ (*p*-value)
Wear gloves before touching membranes and non-intac skin	64.0	82.26	6.833(0.077)
Wear goggles while treating patients	13.73	3.28	9.714(0.046)
Wear protective gowns while treating patients	64.71	72.13	7.932(0.094)
Wear protective masks while treating patients	23.53	66.13	42.556(<0.001)
Discard sharps into a waste container after treating patients	34.69	29.51	1.978(0.740)
Wash hands before and after treating patients	58.82	62.9	1.695(0.428)
Wash hands after contact with patients’ body fluids	88.24	77.05	2.752(0.253)
Take every patient as a contagious disease risk	76.47	95.0	8.143(0.017)

## Discussion

It has been established that HBV infection is a global concern among HCW's and that vaccination is a cardinal point in the prevention of spread of this deadly disease. Since its availability in 1982, it has shown 95% effectiveness in preventing infection, being the first vaccine against cancer [21]. Medical personnel are at a higher risk of contacting the HBV because of their contact with blood and body fluids. The general expectation is that high health literacy level is associated with better health behaviour [22] but health professionals have not always showed a health behavior that is commensurate with their health literacy level [23]. Fatusi et al [24] concluded that they were the most exposed to HBV infection and were the most literate about the infection but unfortunately, they were the least enthusiastic about HBV vaccination. In this study, we evaluated the level of uptake of HBV and practice of standard precaution among clinical medical and dental students at the Obafemi Awolowo University, Ile-Ife, Nigeria. We reported an uptake rate of 83.2% of the participants receiving at least a dose of the vaccine while 95.7% of those that had the vaccine (amounting to 79.6% of the entire participants) completed the three doses. Even though the females tended to complete the three doses, none of the parameters showed a statistically significant difference. Similarly, in a study by Samuel et al [25], females had a higher compliance with HBV vaccination and the difference was statistically significant (p < 0.01). This is in agreement with the observation that women tend to adopt more health-seeking behaviour than men [26].

In a 2007 survey, Simard et al [27] concluded that 75% of HCW's in the United States have been vaccinated against HBV while Habiba et al [28] in a 2011 study reported that 84% of HCW's in Kuwait received three doses of the HBV vaccine. Samuel et al [25] reported that in Southern Nigeria, 70.2% of HCW's have ever received HBV vaccine and only 59.4% received the three doses. In the present study, we recorded a higher vaccine uptake with 83.2% taking at least one dose and 79.6% completing the three doses. Other studies show a low acceptance rate for the HBV vaccine among Nigerian doctors -only 66.3% taking the vaccine [16], 68.6% starting the vaccination while only 20% completing [17] and 50.6% starting the vaccination and only 36.3% completing [18]. Developing nations tend to have low vaccination rates especially among clinical students. The rate of complete vaccination reported in our study is higher than that found among Pakistani medical students which ranged from 42.2% [29] to 70.6% [30]. The HBV vaccination rate is low among the medical students at the University of Port Harcourt, Nigeria: only 34.8% received a complete vaccination. Odusanya et al [20] reported that only 2.6% of the students of the Lagos State College of Medicine, Nigeria received complete vaccination. At the Niger Delta University Teaching Hospital in Bayelsa State, Nigeria, only 19.2% of the medical students had complete HBV vaccination [31]. This is in contrast to a developed nation like Australia. In a survey conducted at the Royal Prince Alfred Hospital, Sydney, 98% of medical students and 95% of dental students received full HBV vaccination. They also reported that students were more likely to be vaccinated than hospital staff (p = 0.001) [19]. This situation of higher uptake among students is similar to what is found in our hospital with a complete vaccination rate of 76.9% among students as reported in this study and 53.8% and 40.3% among the entire staff and the doctors respectively as reported by Fatusi et al [24].

In a Pakistani study, high cost of vaccination was the most often cited reason among HCW's for failing to be vaccinated while belief that they were not at risk was the most dominant reason among medical students [29]. Among dental surgeons in Benin City, Nigeria, lack of opportunity (that is, busy schedule) was the most often cited reason for failing to be vaccinated [17] while the same reason was cited by medical students at the University of Port-Harcourt, Nigeria [31]. This can be related to high pressure of work on the side of the practitioners [24] and academics on the part of the students. Strangely, some studies reported that most HCW's could not cite any reason for failing to be vaccinated [25, 28]. In our study, most students practiced wearing gloves before touching mucous membranes and non-intact skin. This is similar to the studies that reported that most Nigerian HCW's practiced wearing of gloves [25, 32]. In an Iranian study of university-affiliated health institutions, wearing gloves before touching mucous membranes and washing hands after contact with infective materials were the most practiced standard precaution items among HCW's [33]. Singh et al reported that most (61.2%) dental students were not vaccinated against HBV but 95.5% washed their hands before and after examining patients [34].

One of the strengths of this study is the 94.2% response rate. The number of participants was also adequate considering the target population: the total number of clinical medical and dental students ranges from 390-420 in any particular academic session and we were able to recruit 113 participants. The limitations of this study include the potential reporting bias that plagues self-administered questionnaire studies making the participants tend to over-report their compliance. Another limitation includes the heavy imbalance of the distribution of the participants across classes: 95.6% of the participants were final year students and this may call for some caution in interpreting the results of this study. Despite this impressive report, more work needs to be done in ensuring that the vaccination rate rises even higher and approaches the rates recorded in developed nations. There is yet no institutionalized protocol for the vaccination of clinical students in this university and the decision to be vaccinated has been left solely to the initiative of the students. This is similar to the situation in India where the universities do not implement the vaccination policy even though the Indian Dental Council makes it mandatory for all dental students to be vaccinated on matriculation against HBV [34]. With the infection rate which has been estimated to be 13.6% (95% CI: 11.5-15.7%) in the country, the Nigerian government and institutions need to institute more active measures to prevent transmission.

## Conclusion

The vaccination rate recorded in this study is one of the highest in any developing country like Nigeria. Female students had a higher uptake rate just as dental students had a higher uptake rate than their medical counterparts and busy schedule was the most cited reason for not getting vaccinated. High compliance rates with standard precaution were also recorded in the study population with gloves wearing being the most practiced prevention strategy against Hepatitis B virus infection.

### What is known about this topic

- Available reports claim utilization of the prevention strategies (including uptake of hepatitis B vaccine) against hepatitis B infection is low among health professionals despite their literacy level;
- The rate of uptake is much lower in developing countries.

### What this study adds

- A higher uptake of Hepatitis B vaccination is feasible among clinical medical and dental students if they are adequately sensitized;
- The need for institutional protocol for the vaccination of clinical students as a means of ensuring compliance.

## Competing interests

The authors declare no competing interests.
